# Constructing Success: The World Bank, Onchocerciasis Control, and What Lies Beneath Triumphalist Global Health Narratives

**DOI:** 10.1093/shm/hkae064

**Published:** 2025-03-01

**Authors:** Janelle Winters

**Affiliations:** Humanitarian and Conflict Response Institute, University of Manchester, Ellen Wilkinson Building, Manchester M15 6JA, UK; Centre for the History of Science, Technology and Medicine, Faculty of History, University of Oxford, Oxford, UK

**Keywords:** onchocerciasis control, constructivism, metrics production, World Bank, global health history

## Abstract

Using a historical case study of the World Bank and World Health Organization (WHO)’s Onchocerciasis Control Programme (OCP, 1972–2002), I explore how success is conceptualised in global health and why it matters for policy and priority-setting. First, I summarise the ‘dominant’ OCP success narrative that has emerged since the 1980s, which is based on public health, socio-economic and humanitarian justifications for the programme’s effectiveness. Next, I analyse how socio-economic metrics linking the programme’s disease control to increased labour productivity and agricultural land availability evolved in the 1980–90s. This alternative analysis of the OCP demonstrates how metrics, particularly when divorced from their assumptions and political context, are pliable and constructible. I argue that the OCP’s success was *actively constructed* by the World Bank and that moving beyond triumphalist, programme-level ‘lessons-learned’ approaches within global health requires disruption of the epistemic, institutional and discursive power that ‘lies beneath’ success narratives.

When entering the World Health Organization (WHO) in Geneva, visitors pass two bronze statues commemorating the global partnerships for smallpox elimination and onchocerciasis (or ‘river blindness’) control. The latter statue shows a blind man being led by a boy with a stick (Figure 1). This image of a child leading a blind adult (usually a man) dates to at least 1952,^1^ and variations of it were commonly displayed in promotional materials for the Onchocerciasis Control Programme in West Africa (OCP), a regional disease control programme that operated across a swarth of land more than twice the size of France^2^ in eleven countries from 1972 to 2002.^3^Figure 2 shows the most commonly used onchocerciasis photograph, which was displayed on the front of the programme’s preparatory report in 1972.

**Fig. 1.**
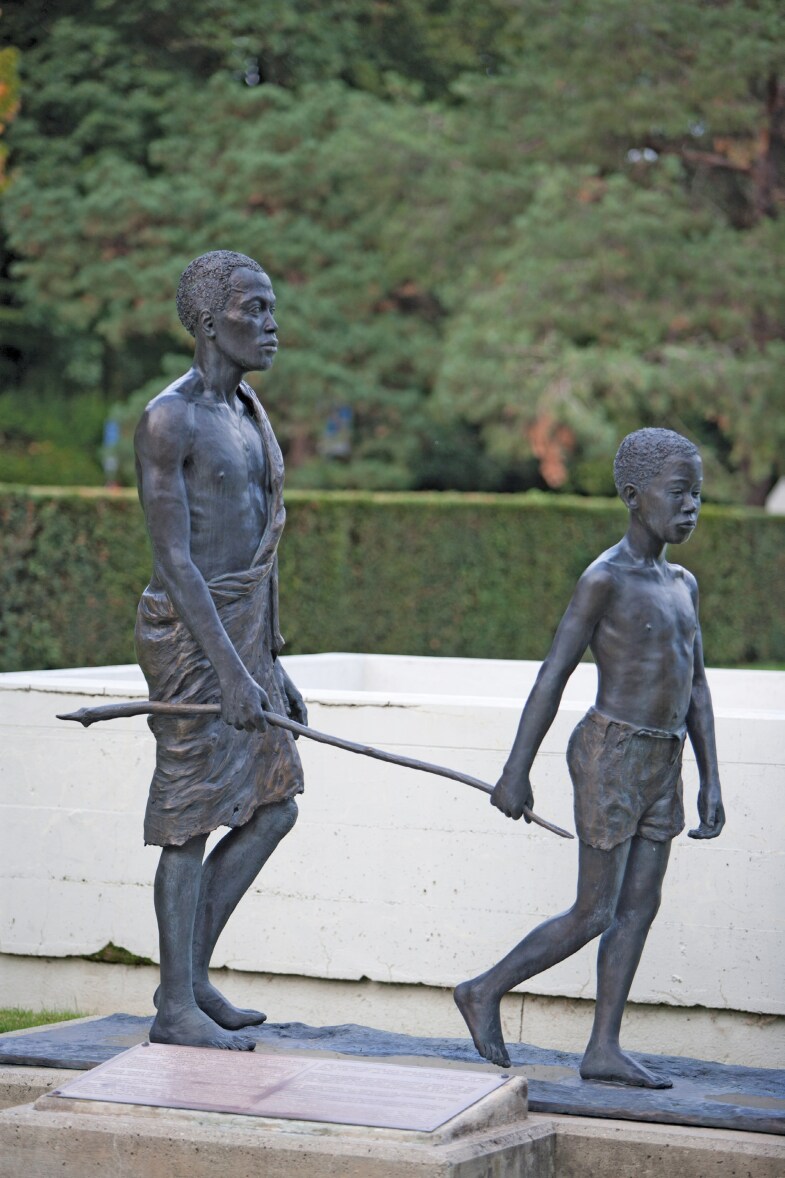
Statue commemorating onchocerciasis control at the WHO Headquarters in Geneva, with the inscription: ‘The statue commemorates the success of three WHO / Pan-American Health Organization-led programmes: the Onchocerciasis Control Programme in West Africa (OCP) operating in eleven countries; the African Programme for Onchocerciasis Control (APOC) covering nineteen countries outside West Africa; and the Onchocerciasis Elimination Program for the Americas (OEPA) present in six countries’. (‘Different views of the Headquarters in Geneva, Switzerland. Statue of an adult blinded by onchocerciasis (river blindness) and guided by a child’, copyright WHO/Christopher Black, 2012. Reproduced with permission from the WHO Photo Library.)

**Fig. 2.**
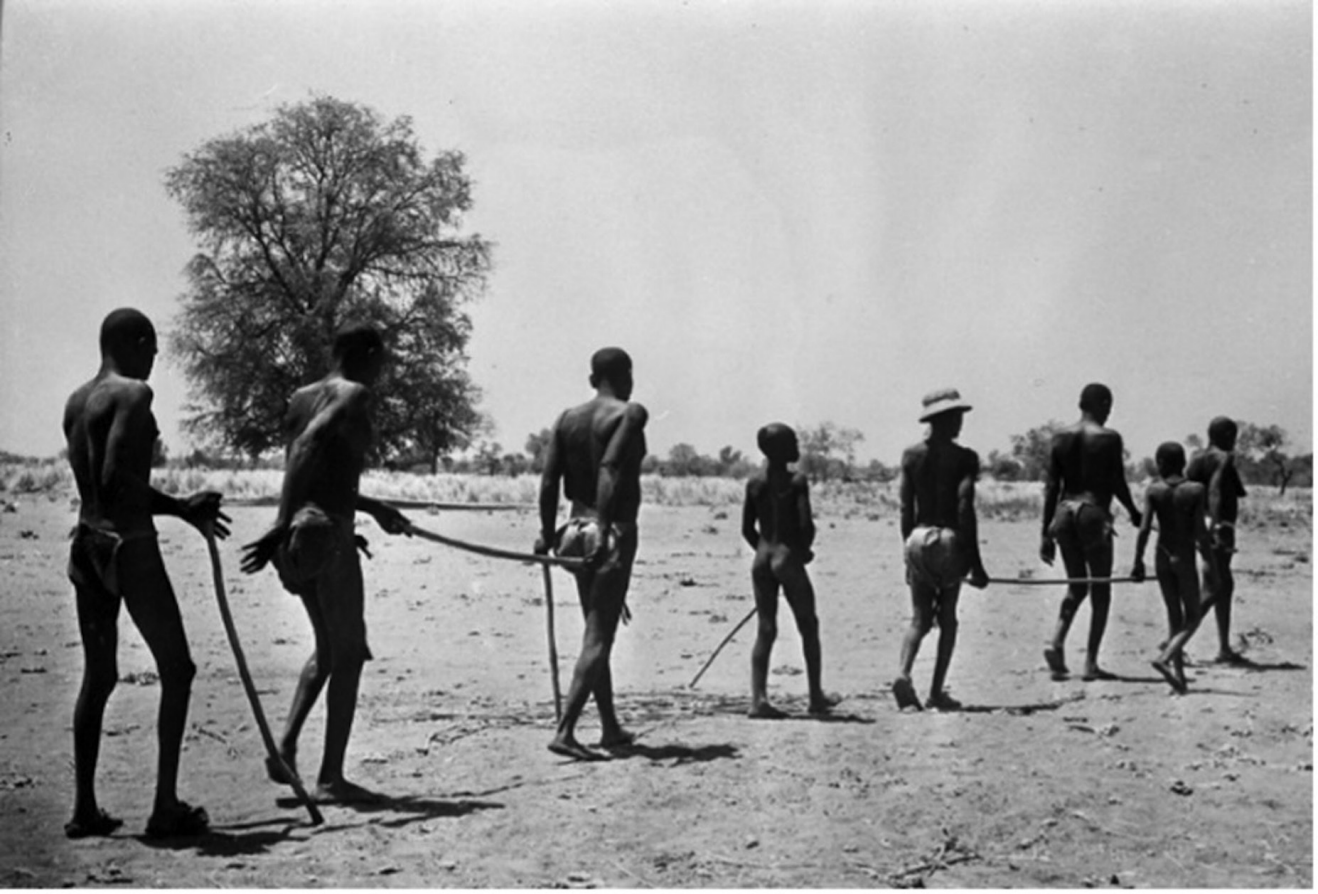
The most famous ‘onchocerciasis image’ used by pharmaceutical companies and in programme publications (‘In many villages the ravages of onchocerciasis are such that one guide has to lead 3 or 4 blind neighbours’, copyright WHO, c. 1970. Reproduced with permission from the WHO Photo Library.)

The OCP sought to control onchocerciasis, a vector-borne disease caused by the roundworm *Onchocerca volvulus* that can lead to blindness and is now classified as one of 21 neglected tropical diseases (NTDs),^4^ and it was sponsored by a unique combination of Bretton Woods and United Nations agencies. Six additional bronze statues are now situated outside institutions of power in global health, including the World Bank, African secretariat of the WHO, pharmaceutical giant Merck & Co, Inc. and Carter Center. The statue in the World Bank’s atrium contains the inscription, ‘Defeating River Blindness in Africa: Successful Human Development Through Global Partnership’.

These statues are emblematic of the long-term position of the OCP and its follow-up programme, the African Programme for Onchocerciasis Control (APOC, 1995–2015), within the collective memory of its executing agency, the WHO, and its fiscal agent, the World Bank. Institutional success narratives have been echoed by the popular press, with hundreds of newspaper and magazine articles^5^ and at least 10 films^6^ showcasing the successes of the OCP since the 1980s. A typical feature story^7^ takes the reader to the riverine ‘onchocerciasis zones’ of the West African Sahel, where blind victims (sometimes called the ‘living dead’)^8^ in rural villages reinforce the disease’s debilitating personal and socio-economic impacts. Rather than dwelling on structural or social determinants of health in these communities, the typical story zooms in on technology and its potential to empower people to help themselves. This technology takes the form of helicopters, which the OCP used to spray insecticides in river valleys to stop disease transmission by killing the black fly vector, and—beginning in the late 1980s—pills of ivermectin (the same drug in dog heartworm pills and made famous by some politicians as a COVID-19 ‘miracle cure’), which were donated by Merck & Co, Inc. and distributed by the OCP to prevent onchocerciasis disease.

While the word ‘success’ and the related word ‘effectiveness’ are used pervasively in global health, rarely are they explicitly defined. What does it mean to be a ‘success’ in global health, and what does this say about the epistemological underpinnings of global health policy and practice? I use a case study of the OCP to explore how success has been defined in global health and why it matters. This case study is based on archival reconstruction, oral histories and programme-related report (grey literature) analysis. Specifically, I consider three inter-related questions. First, in what ways has the OCP been seen as a success and by whom? I take a deeper look at the OCP success narrative itself through a literature review of the rationales cited for success during the programmatic years (1972–2002) and until 2017, and the specific metrics used to justify them. Second, what complexities does the ‘dominant’ OCP success narrative overlook? While this question could be answered from multiple vantage points (e.g., programme justification, financing, multilateral and public-private partnerships, delivery), I focus on an extended historical example of sponsoring agencies’ motivations for launching the OCP and use archival sources to trace how socio-economic metrics were created by the World Bank during the OCP, as well as their often-overlooked assumptions. In doing so, I situate the OCP’s objectives and development of indicators within the broader historiography of the Bank’s role in the economisation of health. Third, what does this OCP ‘underlying narrative’ reveal about how success narratives spread within global health history? I describe how the success narrative was cultivated by the Bank and deliberately operationalised using these metrics and compare the OCP underlying narrative to that outlined by Randall Packard for malaria control in the 1990s.

I conclude with a review of the ways in which success has been defined and conceptualised by other global health historians and researchers, through three frameworks, which I call the impact/evidence-based, marketing and constructivist frameworks. I argue that the operationalisation of specific OCP metrics by the World Bank reflects an entrenched ‘impact-based’ model of global health—but that the OCP’s success narrative is best understood through a constructivist lens. Ultimately, the constructability of the OCP success narrative reveals the complex power structures and epistemologies that shape metrics and ‘lie beneath’ dominant narratives of global health success (and progressive or triumphalist historical lessons-learned approaches).

## Unpacking the ‘Dominant’ OCP Success Narrative

**Fig. 3.**
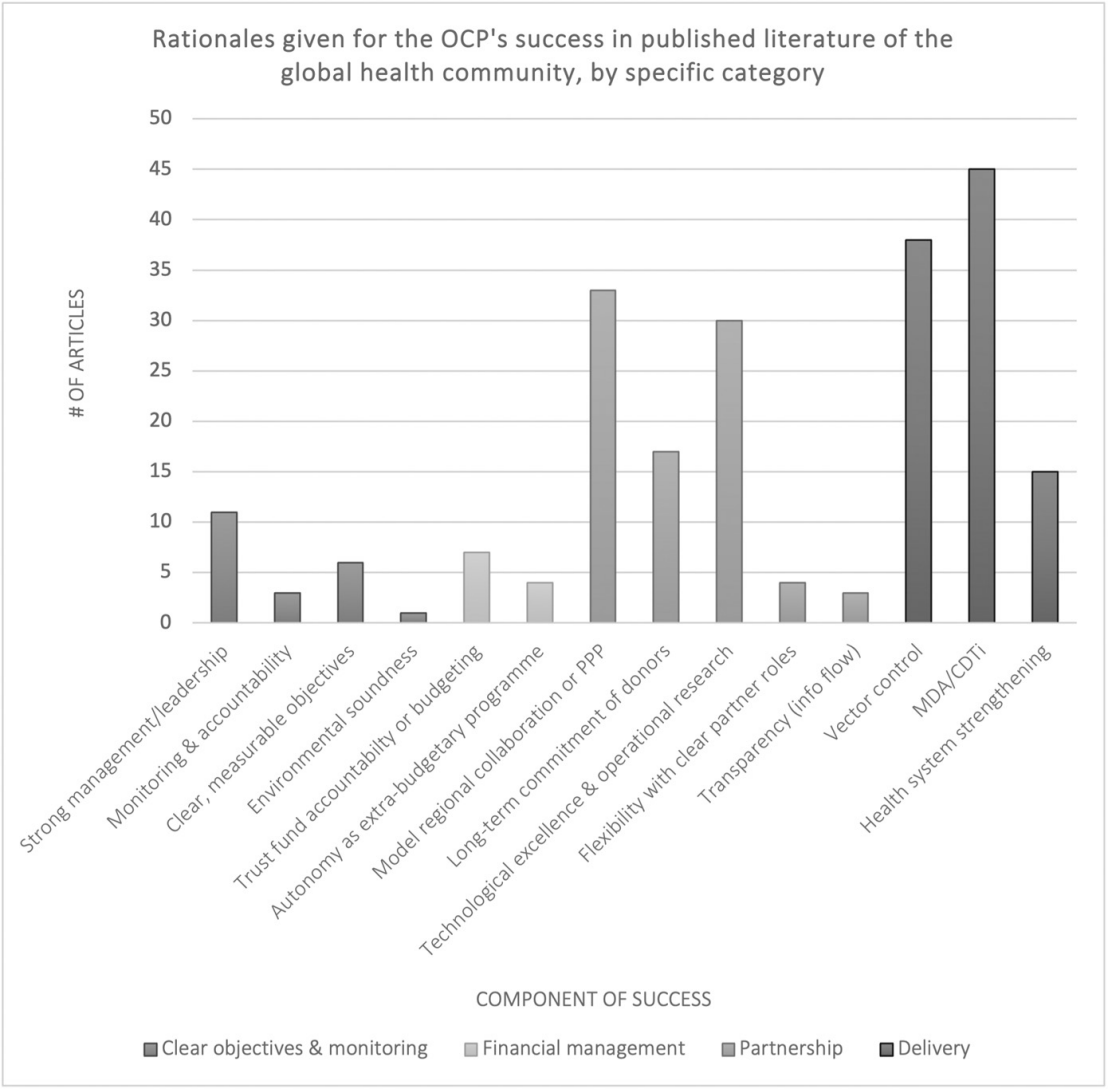
References to success by rationale in publications by the global health community. [MDA is mass-drug administration, PPP is public-private partnership, and CDTi is community-directed treatment with ivermectin.]

A literature review of ‘success’ and the OCP reveals at least 218 publications, including peer-reviewed articles, books and grey literature, that directly refer to the OCP and success from the late 1970s until 2017.^9^ Additionally, over 80 digitised news articles, magazine stories, blogs and websites reference success and the OCP during these years. Aspects of programme success are also discussed in hundreds of memoranda, reports, handwritten notes and draft documents in the World Bank Group Archives. Figure 3 provides a breakdown of the programmatic areas that published sources have identified as contributing to the OCP’s success. These include objectives and monitoring, financing, partnerships and delivery.

**Fig. 4.**
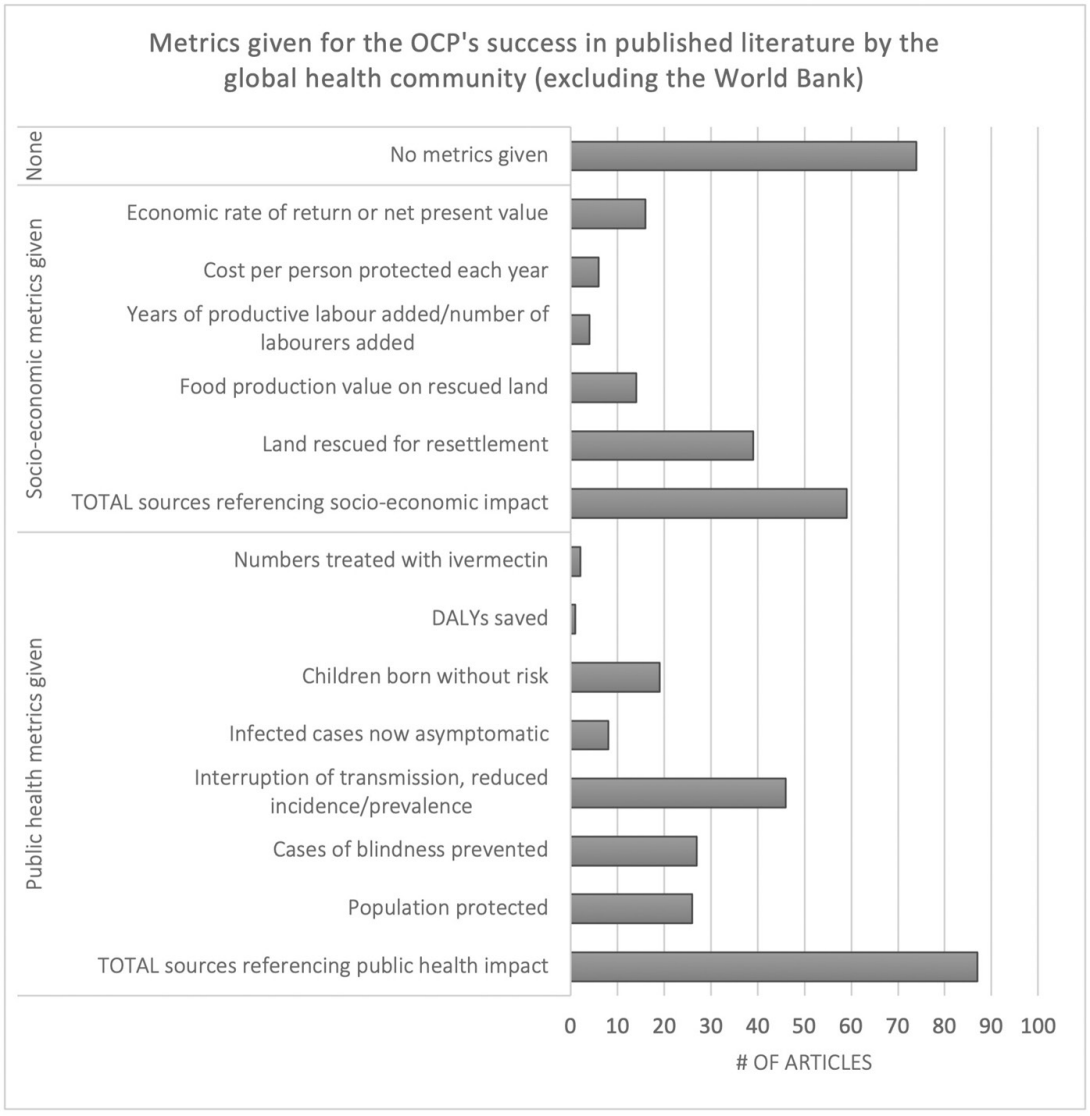
References to success by metric given, in published literature by the global health community. [DALYs are disability-adjusted life years.]

Interestingly, nearly half of all publications that describe the OCP’s success provide no rationale for its success (42 per cent of total publications from 1979 to 2017). This suggests that their authors believe that the OCP’s stature as a success story is self-evident or common knowledge. As shown in Figure 4, the rest rationalise success through public health (49 per cent) or socio-economic metrics (33 per cent), or both. Socio-economic metrics are heavily skewed towards a single metric: the number of hectares of land (25 million) ‘liberated’ for cultivation in controlled zones. Kim and Benton’s cost-effectiveness article (1995), described in the subsequent section, is the most cited of any single source.^10^

**Fig. 5.**
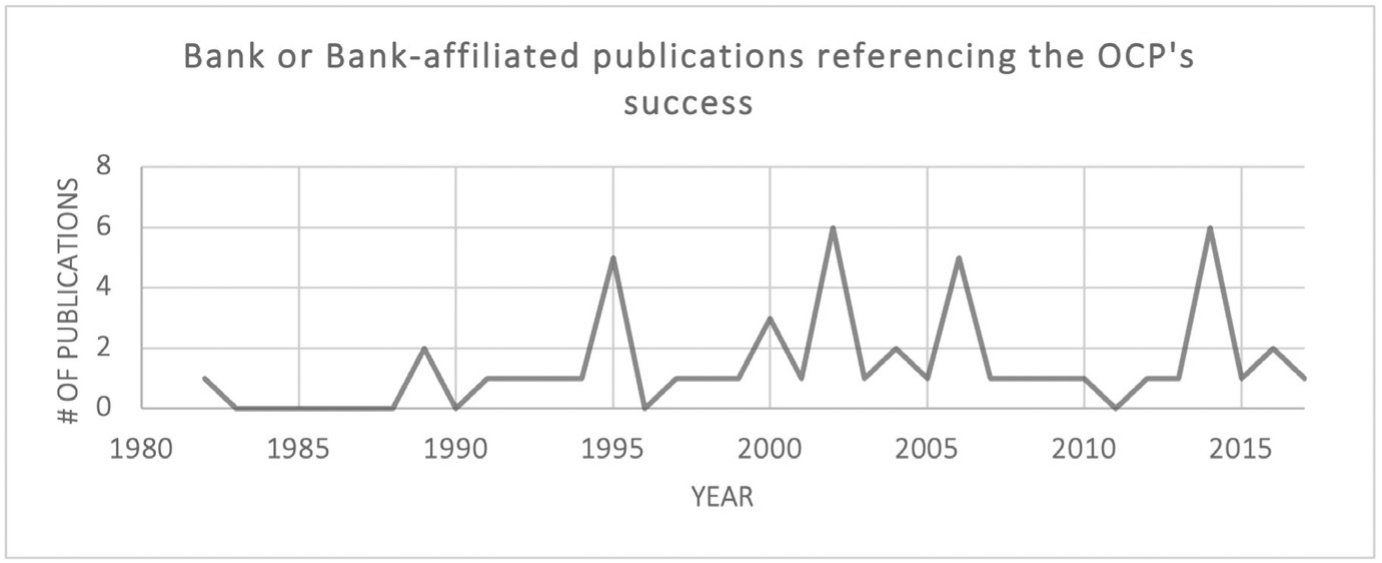
World Bank publications referencing the success of the OCP, by year

**Fig. 6.**
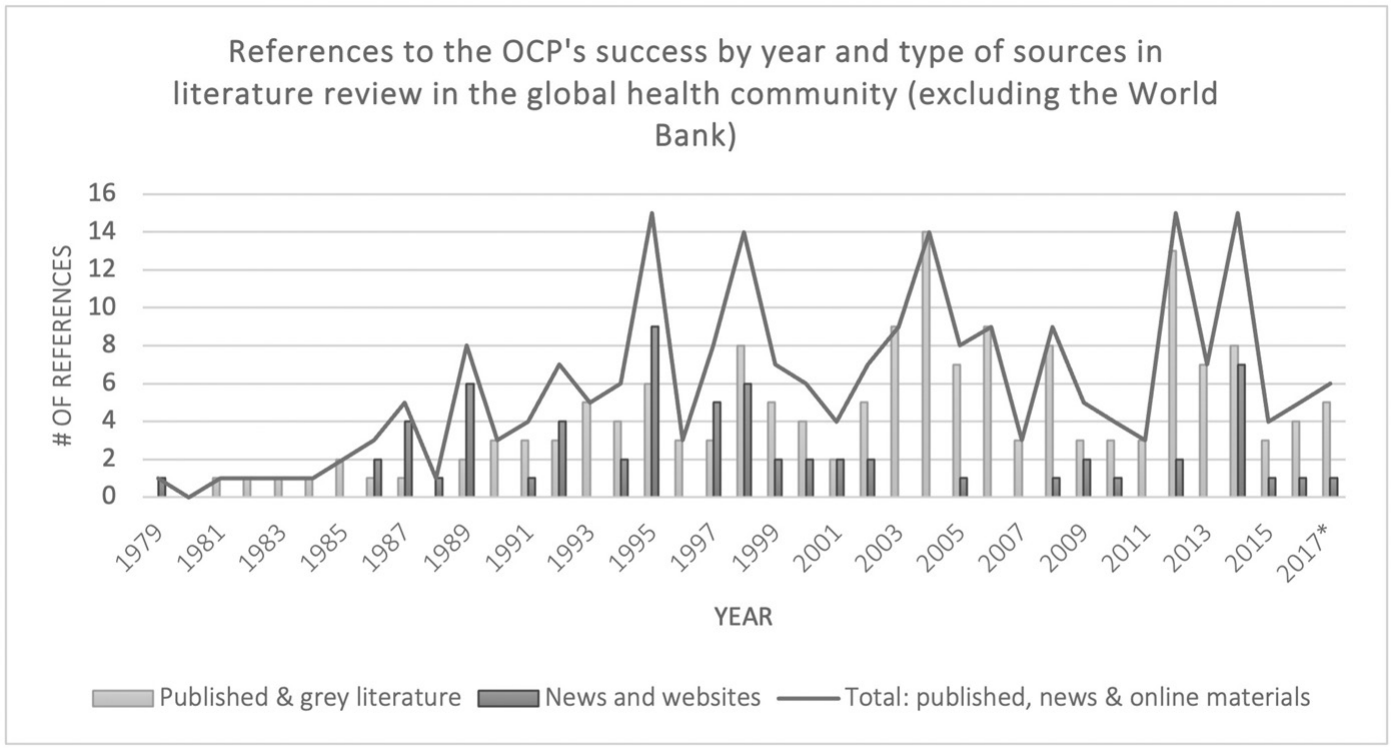
References to the OCP’s success in global health community publications, by year and type of source

This brief window into the dominant OCP narrative shows that the programme’s success has often been taken for granted (i.e., does not appear to warrant a reference, even in academic texts); that success has been rationalised by both socio-economic and health metrics, particularly those produced by the World Bank; and that references to success are often strategically timed. References to programme success did *not* peak when the programme saw positive epidemiological indicators in the mid-1980s. Instead, the first peak in references to the OCP’s success by the Bank was around 1993–5, when the *World Development Report*—which formally linked health to development outcomes—was released and the Bank needed to raise funds for the OCP and APOC in 1995 (Figure 5). The next major peak occurred in 2002, when the OCP was closing and APOC was again fundraising. A third peak occurred after 2012, around the time of the London Declaration for neglected tropical diseases, and the fourth in 2014, at a time of OCP and APOC commemoration (and, crucially, just prior to the launch of the Sustainable Development Goals and the Expanded Special Project for Elimination of Neglected Tropical Diseases [ESPEN] programme).^11^Figure 6 shows references to the OCP’s success by the global health community (excluding the World Bank), which demonstrates a similar trend. Since the OCP ended in 2002, the number of official publications and grey literature that reference its success have increased, although the number of news articles about the OCP have decreased. In other words, the OCP success narrative became more firmly entrenched in the global health community in the mid-1990s and it has remained entrenched to the present day.

## An Underlying OCP Narrative (or What Lies Beneath Metrics)

According to many of the Bank’s descriptions, launching the OCP was a straightforward decision: upon realising that regional development required river blindness control and witnessing the disease’s humanitarian impacts on a trip to West Africa, the Bank’s President Robert McNamara placed it in the hot seat to coordinate its financing.^12^ The Bank’s motivations for investing in the OCP were in reality more complicated. Indeed, onchocerciasis was not an obvious choice as the Bank’s first health investment. It did not pose a health security threat to Western countries; was not well surveyed (geographers postulated that it may be linked to abandonment of river valleys where its vector breeds, but British, French and Belgian colonies had incomplete disease prevalence data)^13^; was not seen as a high priority by most local authorities^14^; and relied on vector control through mass insecticide spraying (a strategy that was widely viewed as unsustainable following the challenges of the WHO’s Global Malaria Eradication programme).^15^

The complex negotiations for the launch of the OCP in the late 1960s and early 1970s are beyond the scope of this paper. In brief, the Bank was at least as motivated in developing onchocerciasis control as a prototype for the economic benefits of disease control as it was in meeting humanitarian goals, and it was especially keen to do so within Africa. Daniel Speich Chassé has described how late British colonial policy 'diagnosed an under-utilised labour force and significant natural resources and called for the development of the exploitation of these potentials’,^16^ and Jesse Bump further explains how such colonial population surveys evolved into regional impulses for disease control. Bump emphasises how geopolitical considerations^17^ (especially the Sahelian drought of the early 1970s and the United States Agency for International Development [USAID]’s interest in West African disease control); the Bank’s burgeoning interest in poverty alleviation and its embrace of a basic needs approach under Robert McNamara^18^; and the interest of the Bank, USAID and some United Nations (UN) agencies in quantifying the link between health and international development outcomes were crucial motivations for its involvement in OCP planning in the late 1960s.^19^ The Bank’s leadership was clear that, ‘under no circumstances could our Bank finance hygiene or public health programmes'.^20^ However, Bank-associated economists *were* interested in formally linking economic development in Africa to health programmes. From 1971-72, the Bank, WHO and United Nations Development Programme (UNDP) held a series of joint meetings ‘in the search for a parasitic disease which could usefully be the subject of model making’, and considered whether onchocerciasis and schistosomiasis could ‘provide a basic methodology for the economic assessment of parasitic disease control programmes of any kind’.^21^ Following these discussions and McNamara’s push,^22^ the Bank’s board decided that it could become involved in onchocerciasis control because improved health would *naturally* lead to improved development outcomes. This marked the Bank’s first grant for a health project; it would not sanction such direct lending for core health sector projects until 1979.^23^ Sponsoring agencies settled on a programme objective in 1975: to *eliminate the disease as an obstacle to socio-economic development in the region concerned*.^24^

How did the Bank measure progress towards this objective over time? I describe six sets of socio-economic metrics, summarised in Table 1, that were developed over two decades amidst continuing uncertainty about how to justify the programme on economic grounds. I argue that uncertainty played out at the Bank in three chronological phases from the 1970s to the early 2000s, which correspond with increasing economization and triumphalism: (1) concern that the ‘fertile valley’ hypothesis was problematic and that land’s intrinsic value for future resettlement may be a bad way of justifying a disease control programme, (2) guarded attempts to quantify both land and labour productivity in ‘onchocerciasis freed zones’ every few years and (3) embracing quantification attempts largely based on estimates rather than data innovations.

**Table 1.** Summary of major OCP socio-economic quantitative studies and the metrics that they put forward

Study	Metric	Value	Major assumptions
Preparatory (PAG) Mission, 1973	Rate of return	10 per cent	General estimate only
	Jobs created	200,000	General estimate only
	Economic of increased industry	$10+ million/year	Due to job creation, general estimate only
	Land freed	65 million hectares	General estimate only
Economic Review Mission, 1978	Net present value (labour)	$20.5 million to $1,137.6 million	25–50 year time horizon, 3–15 per cent discount rate, $45–70 income per worker, 3 per cent growth in income/year
	Land freed	5.06 million hectares (initial), 13.4–15.4 million hectares (revised)	For seven initial OCP countries
Prost & Prescott, 1983	Years of productive healthy life added	1,098,094 productive health years of life added, 147,291 discounted years of productive healthy life added	One case of blindness reduces productive life by 20 years in mesoendemic areas and 23 years in hyperendemic areas, 10 per cent discount rate, 20 year time horizon
	Cost per productive year of healthy life added	$20/year of productive healthy life, $150/discounted year of productive healthy life	20 year time horizon
USAID Impact Review, 1986	Land freed	15 million hectares	None given
	Productive value of land	Can feed 10 million/year	Land available for harvest is planted entirely with sorghum, 2.5 million hectares of land is available to harvest each year, 2 million extra tons of grain produced/year
Younger & Zongo, 1989	Rate of return (land & labour)	−6 to 69 per cent	25–50 year time horizon, 5–15 per cent discount rate, $57–107 income per worker, 1–3 per cent growth in income/year, $10–30/worker land differential
	Land freed	9.9 million or 18.2 million hectares	Economic Review Mission’s range of land in seven countries (5.06–13.4 million hectares), plus a loose Bank estimate of land in southern/western extensions (4.84 million hectares)
	Net present value (land)	$−123 million to $315 million	(Same as rate of return)
	Net present value (labour)	$30 million to $1,137 million	(Same as rate of return)
Benton & Skinner, 1989	Rate of return (land and labour)	11 –13 per cent	50 year time horizon, 15 million hectares of land (only) cleared, 3 per cent land settled each year, Workers plant 3–4 hectares each, $10/worker land differential, 5–10 per cent discount rate,
	Rate of return (labour only)	63 per cent	Costs from 1990 to 2004 (excluding ‘sunken costs’ and counting costs from the present), 5 per cent discount rate
	Land freed	25 million hectares	15 million hectares from original 7 countries, 5 million Southern extension, 5 million Western extension
	Productive value of land	Can feed 17 million/year	Same methodology of 1986 impact review, Assumes all land is freed of other agricultural constraints
	Cost per person protected	$0.54/year	11 country area, Population total is 25.9 million
	Cost per at-risk person protected	$0.45/years	About 50 per cent of total population in countries at risk, Measures protection over 50 years
Kim & Benton 1995	Rate of return (land and labour)	18–21 per cent	29–39 year time horizon, 3–10 per cent discount rate, 70–100 per cent labour force participation and land utilization, 1.7 per cent population growth/year, Each case of blindness prevents 20 years of productive life, 0.66 output elasticity of labour
	Net present value	$485 million to $3,729 million	39 year time horizon, 3–10 per cent discount rate, 85 per cent labour force participation and land utilization, Constant 1987 USD
	Cost per person protected	$0.57/year	Across 11 countries, Constant 1987 USD

*Phase 1*. At the launch of the OCP, an estimated 65 million hectares of land were viewed as abandoned due to river blindness prevalence and therefore available for post-control resettlement. This area was thought to provide ‘some of the best land’ with ‘good soils’^25^ and more plentiful rainfall than the ‘old lands’.^26^ Such characteristics made it a ‘major new resource’^27^ in a region recently devastated by drought and poor food security. In agreeing to package the OCP into a single objective, with a primary focus on ending disease transmission and a secondary focus on fostering socio-economic development in the onchocerciasis control area, sponsoring agencies were essentially buying time to develop economic models that could capture the benefits of onchocerciasis control. In doing so, they were able to make future promises to meet the demands of donors like Canada, the United States and France, who emphasised the critical importance that they placed on economic development.^28^ The Bank estimated that the investment potential for the onchocerciasis control area over 20 years was approximately $250 million, or $64 million from 1974 to 1979.^29^ This investment potential was based on general land surveys and the concept of human capital, or the evolving idea in the 1970s that the human body is a productive resource that can be invested in and stockpiled for future individual and societal use.^30^

Within the first few years of the OCP’s launch, there was ‘considerable confusion’ among donors and sponsoring agencies about how to develop valleys freed from onchocerciasis due to vector control operations, at least in part because most of the seven original participating West African governments lacked the ministerial capacity to prepare development plans for the lands and coordinate funding for them.^31^ The Bank’s OCP development methodology faced challenges on three fronts. The first was ideological, reflecting a worry that it was too focused on ambitious future development plans at the expense of guiding spontaneous resettlement. The second was that the control area was based on entomological patterns—black fly (the disease vector) distribution—not economic patterns.^32^ This made national buy-in to regionally integrated development plans complicated.^33^ For example, dramatic population pressures outside of OCP lands meant that the Upper Volta government gave a high priority to formally settling onchocerciasis-freed zones, while Ghana and Niger typically had less population pressure outside of control areas and were loath to support formal planned settlement. The third area was the relative prioritization to assign to onchocerciasis control lands. Was it possible—or ethical—to demand that countries preferentially develop onchocerciasis freed lands over other lands, because donors had invested in a disease-control programme?^34^ Added to these complex socio-economic development planning dilemmas was the growing understanding that some of the OCP lands might not even be candidates for human (re)settlement. Riverain land would need to be reserved for dams, forest or game grazing and water storage, to prevent ecological destruction and poor future agricultural yields.^35^

By 1977, the general sentiment in the Bank’s West Africa department was that measuring the effects of socio-economic development in OCP areas was *much* more complicated than initially expected. Would labour or land represent the major measurable economic benefits of OCP? What data was absolutely required to model socio-economic benefits of the programme, and how could economists account for other socio-economic variables, like migration patterns, trypanosomiasis (sleeping sickness disease) and water availability? A speaker at a 1977 panel lamented that the Ouagadougou OCP secretariat ‘offered a perfect microcosm of what global inter-agency non-cooperation was like’, and Bank staff internally declared ‘we believe that the economic argument for the oncho problem has a weak basis’. Bank staff proposed an Economic Review Mission, to be undertaken in 1978, which would ‘test the fertile valley myth’ once and for all.^36^

The Economic Review Mission did more to inflame than quell the OCP’s economic controversies. The Bank financed most of the mission, and nominated Elliot Berg, a professor and economist from Michigan State University, to coordinate it.^37^ Berg would later author an influential 1981 report that provided an impetus for structural adjustment, *Accelerated Development in Sub-Saharan Africa,* commonly referred to as the ‘Berg report’.^38^

The mission evaluated two types of economic benefits: direct (improved productivity of labour) and indirect (opening of new lands).^39^ For the labour-related benefits, the present value of estimated OCP costs was compared with the present value of productivity gains. Berg’s team found that the net present value of labour-related benefits varied dramatically—from $20.5 million to $1.14 billion in constant 1978 USD—depending on values used for the model’s variables. These variables included the time horizon (25 or 50 years), discount rate (3 or 10 per cent), and estimate of the productivity of labourers (the marginal product of labour, which measures the change in labour output due to blindness). Discount rates are coefficients used by economists to account for the fact that, from a policy perspective, lives saved in the immediate future are typically considered more valuable than those in the more distant future (higher rates prioritise future populations more and generally reduce the estimated immediate financial benefits for a programme). The value of labour benefits only approached the OCP’s estimated costs using favourable variables: a long (50-year) time horizon, a modest discount rate (10 per cent) and the highest marginal output of labour estimate.^40^ The first version of the review mission’s report also dramatically reduced the estimates of productive land that would be made available through the OCP. Instead of considering all land in the original OCP area as productive, the original report estimated that only 5.06 million hectares would be suitable for cultivation.^41^

The report was heavily criticised by nearly all OCP partners.^42^ Despite the general consensus that the report ‘underestimated the economic benefits and was pessimistic’, the panel and seven participating countries ultimately endorsed its final version. This version provided a much higher estimate of the arable land that could be made available by the OCP in the seven original countries, with little substantiation: 13.4–15.4 million hectares.^43^ Berg responded to harsh criticism with a general attack on the idea that ‘new lands’ could form a justification for health programme investment at all. An FAO representative paraphrased Berg’s argument:

*There was a state of the art in carrying out this type of evaluation: in justifying health projects, the humanitarian objective is most important. Economic evaluations are based on the labour available effect of the project, how it can increase labour and therefore production, if the people do not die… Economists, he said, have not yet come to grips with the ‘new land’ effects of such projects. He said that there was no economic justification in saying that because we had spent a lot of money on the control of oncho, we must therefore validate the investment by further investment in the area.* ^44^

This period marked a significant shift away from the Bank’s earlier focus on classical economics for health care analyses. The *Accelerated Development* report laid out a neoliberal agenda for health financing, which was gradually institutionalised through Bank economists. Historian Howard Stein has argued that, by 1985, these economists’ reports positioned principles of efficiency, affordability and effectiveness as primary determinants of health sector decision-making, contributing to its push of user fees, privatisation and decentralisation of health services.^45^

*Phase 2*. During this context in the mid-1980s, a series of fresh attempts were made to measure and model the OCP’s socio-economic progress. These attempts continued to lack precise data for most of their variables. By 1990, language about the economic rate of return and the number of hectares ‘freed’ for cultivation were widely used, both by OCP sponsoring agencies and in secondary literature, but the programme continued to invest very little in socio-economic development planning or projects.

In the early 1980s, as some donors worried that ‘the economic development justification for the program had been swept under the rug’,^46^ the Bank reinforced that the ‘OCP should not become an economic development agency, but should supply the beneficiary countries and donors with basic indicators (like land availability and degree of resettlement) measuring the results of the 10 years of disease control’.^47^ Around this time, two economists working out of the Bank’s Population, Health and Nutrition (PHN) department attempted to do just this.

These economists considered more complex economic questions than their predecessors: What alternatives (variants) should be used in OCP cost-effectiveness or cost-benefit calculations? What is a reasonable rate of return to justify continuation of a health programme? One of these Bank economists, Nicholas Prescott, argued that ‘if no feasible variant has net social benefits larger than zero, then continuation of the programme would be rejected’.^48^ Prescott was eschewing Bank involvement for purely humanitarian investments, and suggesting that only a sound economic study would legitimate the OCP’s continuation, although he admitted that if the OCP was deemed not cost-effective compared to other potential health interventions in the region it might still have to continue for political reasons.

As Anne Mills has outlined, such cost-benefit analyses—which compare the numerical costs of two or more programmes or programme variants—were first applied to public sector investment decisions in the 1950–60s. They increased in frequency during the 1970s for health programme justification, although they were typically 'extremely crude and showed only a passing acquaintance with empirical evidence' until the 1970s.^49^ In the case of the OCP, a cost-benefit analysis could help the WHO and Bank choose the programme conditions (variants) with the greatest net benefit, while a cost-effectiveness analysis was generally seen as less optimal for internal programme decision-making (since it would use a standard indicator to compare the programme’s benefit to an alternative health programme).^50^

Interestingly, when Prescott and his colleague at the Bank, Andre Prost, performed their economic analysis of the OCP, they chose to undertake a cost-effectiveness (not a cost-benefit) analysis with no different programme variants as comparators and focused on only one country (Upper Volta). They chalked this choice, which severely limited the policy relevance of their study, to the ‘practical difficulty of undertaking a comprehensive and empirically defensible assessment of all the benefits of onchocerciasis control’.^51^ Prost and Prescott chose to use the metric ‘healthy years of life’ for their analysis. This metric was put forward as a ‘health status index model’ in 1976, was first used in a developing country context in 1981 and can be seen in many ways as a precursor to the now predominant ‘disability-adjusted life years’ (DALYs) metric (a major difference is that healthy years of life are maximised for effective programmes, while DALYs are minimised).^52^ In the case of onchocerciasis, healthy years of life added together two figures: the values of preventing disability and postponing death. For the purposes of the study, blindness was taken to be fully disabling—one year of blindness was the equivalent of one year of death—although the authors considered that this *underestimated* its impact because blind people could be an economic burden on the household and community, in addition to being unable to productively work. Prost and Prescott then limited their estimate of years of healthy life added to the (economically) productive population and weighed their productive years of healthy life added results by time preference. Finally, Prost and Prescott compared the 20-year estimated costs of the OCP to the productive healthy years of life added by the OCP in the Upper Volta. The total productive years of healthy life added across 20 years of the OCP corresponded to a cost of around $20 per year of productive healthy year of life added or $150 per discounted productive year of healthy life added. Prost and Prescott then compared the OCP in Upper Volta to another health sector project—measles vaccination in the Ivory Coast.^53^ They justified this clearly problematic comparator by simply stating that there was no available data about costs for alternative health projects in Upper Volta. One of the major take-home points of the study sounds intuitive but is often under-appreciated within global health policy and planning: the authors stated that ‘the relative cost-effectiveness of onchocerciasis control is very sensitive to the choice of effectiveness measure’.^54^ The choice to weigh by productive years, time period considered and discount rate used could each *completely determine* whether the OCP or measles vaccination was more cost-effective.

Prost and Prescott’s study did little to quell donors’ concern about socio-economic justifications in 1984–85.^55^ In 1986, a USAID impact review concluded that the OCP was a successful multi-donor initiative, based on public health endpoints (i.e., 90 per cent of transmission interrupted). Yet, it also cautioned that ‘this will not automatically lead to enhanced incomes and economic growth’ unless targeted investment was made in the liberated lands.^56^ The review also provided the first estimate of the total potential output of the onchocerciasis freed lands. It performed a basic calculation of this output based on the Economic Review Mission’s (contested) estimates that around 15 million hectares of new land would be suitable for agricultural purposes in the seven original OCP countries.^57^ If one assumed that settlers would continue to use traditional technology for tilling this land and that each plot of land would be tilled every six years, about 2.5 million hectares were available for cultivation each year, and about 2 million tons of sorghum could be grown on these lands. The OCP new lands could therefore theoretically meet the caloric needs of about 10 million people (or one million families) annually if they ate sorghum alone.^58^ The report underscored that this production potential was *hypothetical* and not yet reflected in settlement or land usage. Thus, the major economic benefit of the OCP would be based on land use (not labour productivity), *if* (and only if) resettlement increased at an adequate rate.

In the mid-1980s, the new onchocerciasis coordinator at the Bank took the lead in coordinating a socio-economic development study, designed to provide better data.^59^ Amidst the OCP’s financial insecurity in 1989, two economists then attempted a fresh cost-benefit analysis. Stephen Younger, an economist from Williams College, and Jean-Baptiste Zongo, a member of the OCP’s Economic Development Unit, decided to recalculate the 1978 Mission’s estimate of productivity gains. They also developed a formula for estimating the net present value of OCP lands.^60^

A major complication continued to be that there was no widely accepted standard to use for many variables that were critical for a cost-benefit analysis of a health programme. For instance, over what time period should the benefits be tracked and compared to costs? If the OCP eliminated the black fly vector, farmers might still be protected from blindness in a millennium. Should benefits be tracked only until the end of the programme? For 50 years? A century? Would it be fair to track benefits far into the future, without adding in costs for maintenance of vector control and other programme activities (which were also not easy to quantify)? And, when tracking the programme benefits over time, what discount rate should be used? Would a low (three per cent) discount rate be appropriate, considering that this was a social investment programme that benefitted the poor rural people, or was a much higher discount rate (10–15 per cent), as traditionally used in analysis of the Bank’s infrastructure projects, appropriate for comparison purposes? Furthermore, were the estimates of value of labour output per worker accurate, especially since they were extrapolated from small country-based studies and expanded across the whole OCP area? Did using low output of labour values inherently bias health sector allocation against the rural poor, precisely when organizations like the Bank wanted to target them with development opportunities?

The importance of these questions goes to the heart of Younger and Zongo’s findings. They began their study with the caveat that, as for most health projects, ‘data are not good’ and any internal rate of return would by nature be ‘imprecise’. Indeed, their internal rates of return varied dramatically. Again, for labour increases due to the prevention of blindness, the rate of return was heavily dependent on the time period, value of output per worker and discount rate used. Only the more favourable scenarios made the OCP’s labour benefits surpass its costs.^61^ Based on these findings, Younger and Zongo argued that land value was the ‘most important economic impact that the OCP is likely to have’. This departed from earlier predictions that labour productivity would be the most economically valuable output of the OCP. However, there was still far from a consensus on the amount of fertile land made available by the OCP. Even if the OCP’s planned land settlement review could help to clarify uncertainty about the amount of fertile land made available, a key question also remained: how could one quantify the worth of this land compared to other currently cultivated land, across the wide sweep of 11 countries? What extra, intrinsic value did the OCP land have? Younger and Zongo used both the more conservative and optimistic new land estimates from the 1978 Economic Review Mission and added in the estimated amount of fertile new land from the OCP’s later extensions, for a total of 9.9–18.2 million hectares. Because no data was available on the market value of this land, the authors extrapolated a ‘land differential’ (the added benefit of this land) from data about planned settlement in Upper Volta alone. They further reasoned that it was not reasonable to assume that all lands would be instantly resettled, so they used data from a 1984 study in the same (single) country^62^ to estimate that about 1.62 per cent of the lands available would be resettled each year. No socio-economic data from other OCP countries was used to calculate the land benefits. The major factor in whether land benefits outweighed costs was, unsurprisingly, the land differential value used. The rate of return for land value varied from about 5 to 10 per cent.

When land and labour benefits were taken together, the rate of return varied from *negative six to 69 per cent* over 25 years and *nine to 69 per cent* over 50 years. Younger and Zongo concluded cryptically that, based on these numbers, ‘At worst, the OCP cannot be viewed as an economic disaster. At best, it has a high rate of return’. Yonger and Zongo reinforced that their wide range in rate of return estimates ‘is common for health projects in developing countries because of the great uncertainty about many key parameters’.^63^

Staff from the Bank’s onchocerciasis unit published a paper on the socio-economic benefits of the OCP in 1989.^64^ Like Younger and Zongo, these staff (Bruce Benton and Elizabeth Skinner) found that the OCP’s rate of return due to labour productivity depended heavily on the values chosen for each variable. Over the course of 50 years, an advantageous (5 per cent) discount rate yielded $338 million in benefits and $231 million in costs, while a more conservative 10 per cent discount rate had higher costs than benefits.^65^

For the land-related benefits, Benton and Skinner put forward the widely cited figure that 25 million hectares of land would be freed for cultivation, or enough to feed about 17 million people each year. This figure was, again, a general estimate, based largely on the contested 1978 Economic Review Mission.^66^ This estimate would not be reproducible by external researchers. Using the methodology from the USAID 1986 impact review evaluation, Benton and Skinner estimated that these hectares of tillable land could eventually produce 3.3 million tons of sorghum per year, or enough to theoretically feed 16.7 million people.^67^ Taken together, labour and land benefits yielded a ‘respectable’ 11–13 per cent rate of return over 50 years. Thus, the 25 million hectares metric was based on broad generalizations, rather than land settlement or agricultural production data from contemporary land surveys. Benton and Skinner admitted that 'these estimates are, at best, rough orders of the magnitude of the cultivatable land expected to be made available largely by oncho control’.^68^ The figures represented the *maximum* realistic amount of land that would reasonably be resettled and cultivated after vector control operations ended, not the land that was *actually* settled or cultivated in 1990, and assumed that there would be no upkeep costs after the end of the programme.

In 1990, the Bank funded a USAID-executed external review of the OCP. This review report stated that the full potential of the OCP-freed lands had not been widely realised; capacity for economic development of these areas was low in every country; and onchocerciasis zone-specific plans had not been effective. It underscored that ‘responsible investments should not be undertaken just because certain areas are onchocerciasis-freed’.^69^ In effect, the ‘hectares freed’ metric distanced donors and sponsoring agencies from responsibility for socio-economic development, delineating national planning and investment allocation as a responsibility of African governments.^70^

*Phase 3.* In many ways, the World Bank’s 1993 *World Development Report* represented a culmination of what Vincanne Adams has deemed a late nineteenth century-initiated quest for ‘a metric that can serve as a universal standard…or what might be called *one metric to rule them all*’.^71^ This metric was the DALY. Stein has argued that the introduction of the DALY was a fulfilment of the Bank’s ‘neoclassically inspired vision of improving the effectiveness of health outcomes by optimizing the health of individuals, subject to resource constraints’,^72^ and Katherine Kenny presents the metric as a way of institutionalising the concept of human capital. In Kenny’s words, this *economisation of health* is accomplished by ‘imagining health as a form of human capital…inseparable from the rising prominence of the World Bank in world health affairs since the late 1980s, its efforts to restructure health systems according to cost-effectiveness, and its greater valuing of the economically productive middle years of life so as to prioritise health for economic growth’.^73^ While the OCP’s metrics continued to include land value calculations in the 1990s (which are not part of the DALY) and little changed in terms of the assumptions underpinning these metrics, Bank staff evoked an increasingly triumphant stance that aligned with such economisation of health from the early 1990s.

As the Bank and WHO discussed broadening ivermectin distribution through a new Pan-African Onchocerciasis Programme—what would later be called APOC (which delivered drugs for onchocerciasis prophylaxis)—in 1994, they pointed to the socio-economic feats of the OCP. Benton underscored the Bank’s success in modelling socio-economic benefits of the programme at a major 1994 donor’s conference. In Benton’s presentation, he stated that the OCP was decidedly a humanitarian success, but that ‘reduced pain and suffering are impossible to quantify’. He then boldly declared the success of the programme socio-economically, in quantitative terms: ‘Until recently, we have had to rely on anecdotal evidence. Now, we have the required data’.^74^

Kim and Benton’s new analysis, published in 1995, directly related inputs (labour available) to economic outputs.^75^ In total, this yielded labour-related benefits over the length of the programme of about $192 million at a low discount rate (3 per cent). At a higher discount rate (10 per cent), the net present value became negative, which would indicate that the programme was unjustifiable from an economic lens. Again, the effects of the OCP on the labour supply and productivity outputs could be entirely reversed, depending on a single variable: the discount rate used. To calculate the land-related benefits, Kim and Benton had a major assumption: that the utilisation of new lands would increase systematically over time.^76^ This was projected from land settlement review data and assumed that *all* land in the original OCP area would be largely used by 2003 (and land in the extension areas by 2014). Based on this logic, Kim and Benton calculated that, from 1972 to 2002, the net present value for land use was $380 million to $3.154 billion. Taken together, Kim and Benton therefore estimated that labour and land benefits over 39 years would yield a net present value of $485 million to $3.729 billion, or a high 18–20 per cent rate of return. The high end of this rate of return is the rate most cited in global health literature. Their analyses indicated that the major economic benefit of the OCP would be derived from land (not labour).

Soon after Kim and Benton’s 1995 paper was published, donors pledged for APOC and the OCP secured funding for its final fiscal phase. A few scholars, like Tim Evans, then a professor at Harvard and later the Director of the Bank’s Health, Nutrition and Population global practice, published short pieces on specific aspects of OCP economics.^77^ However, the Bank and sponsoring agencies’ direct involvement in onchocerciasis area economic development largely subsided.

## Constructing the OCP’s Success

**Fig. 7.**
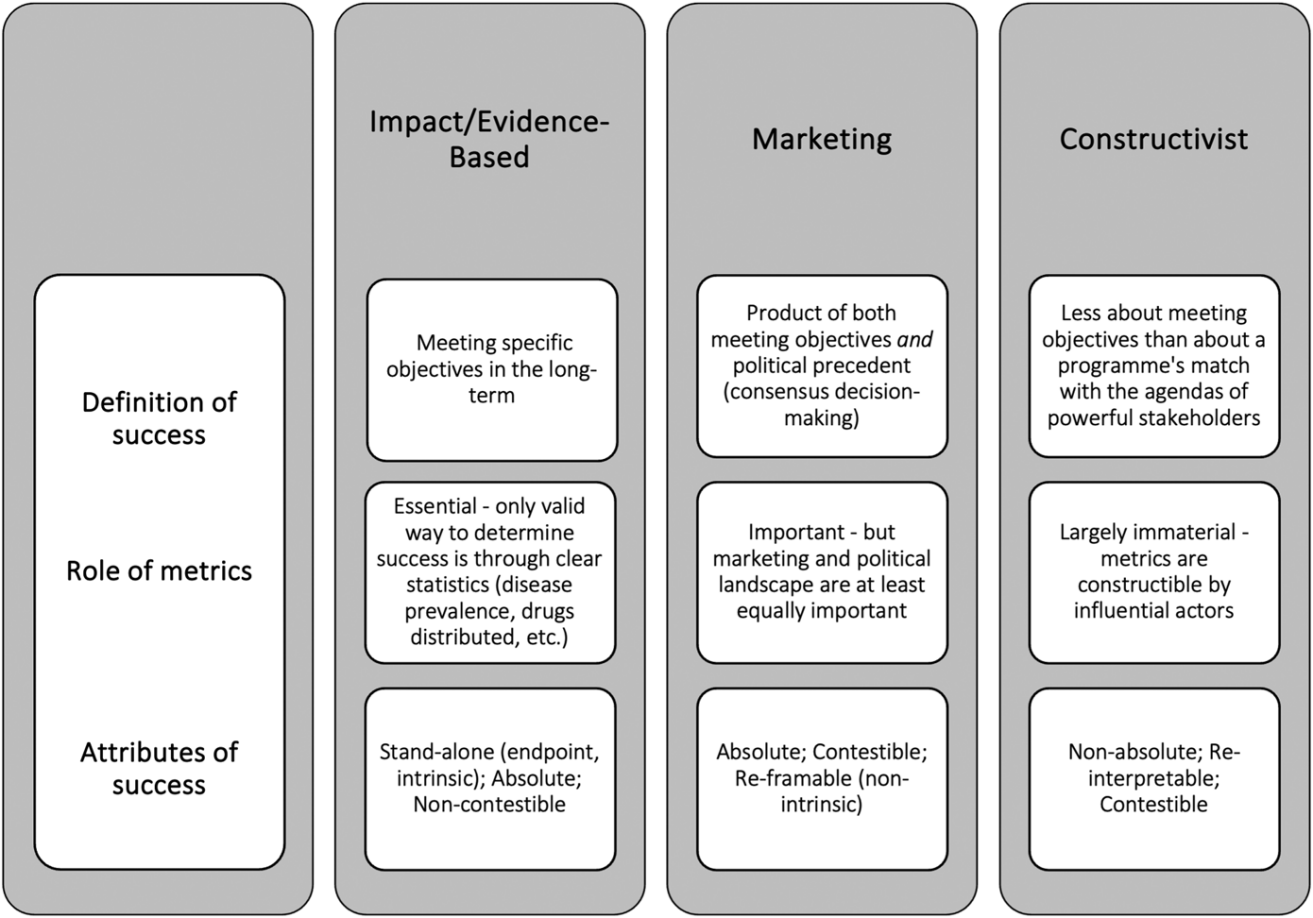
Definitions and attributes of the three theoretical frameworks for defining and understanding success in global health

Based on this case study of the OCP, Anne-Emanuelle Birn’s historical conceptualisation of periods of success in global health,^78^ Suerie Moon’s typographies of success in global health governance,^79^ Marlee Tichenor et al.’s methodological suggestions for interrogating power and global health at the World Bank^80^ and a broader review of global health governance research about success and effectiveness, I propose three frameworks to capture how success has been understood in global health through a historical lens. I define these frameworks as ‘impact/evidence-based’, ‘marketing’ and ‘constructivist’. Figure 7 provides an overview of each of these frameworks, which are best viewed on a spectrum. The three frameworks are designed to capture the way that metrics are shaped, moulded and marshalled to reinforce particular ideologies of health and priority-setting.

*The impact/evidence-based framework:* This framework defines success as the reduced prevalence of disease or the improvement of specific health outcomes, for the largest population possible (taking a rather utilitarian approach). Proponents of this framework place emphasis on technically-grounded indicators—metrics—of success, such as reduced DALYs, cost-effectiveness and level of distribution (e.g., number of drugs or bed nets distributed) for the health intervention. Success is seen as an endpoint; it is the meeting of a programme’s objectives over time through specific indicators, on a sufficiently large scale. As such, the use of specific metrics provides a specific point that can be reached at which a programme becomes a success (such as an evaluation showing improvement of health outcomes). A declaration of success is evidence-based and largely non-contestable.

Since 2000, the Bill & Melinda Gates Foundation has been one of the major supporters of the impact framework. In 2009, it launched a Living Proof Project Campaign to showcase ‘extraordinary success through stories of U.S. involvement in global health’. This awareness campaign used specific metrics to identify and showcase global health successes.^81^ The Gates Foundation also funded a major initiative at the Center for Global Development, the What’s Worked Working Group, which identified large-scale global health success stories from 2002 to 2016.^82^ This working group was composed of 15 international health experts, development economists and public policy experts, approximately half of whom were former Bank employees.^83^ Based on the recommendations of this group, the Center for Global Development released three editions of a book, *Millions Saved*, in 2004, 2007 and 2016.^84^ In the first two iterations of *Millions Saved*, success was defined in terms of impact and a data-driven approach was used to measure impact through precise metrics. For example, the five criteria for success included scale, importance, impact, duration and cost-effectiveness.^85^ The success stories selected were heavily skewed towards vertical programmes, whose impact could be measured through these tight statistics, and included the OCP and APOC.^86^ Bill Gates wrote the forward for the 2016 edition of *Millions Saved*, which showcased 22 new success stories—and four ‘disappointments’. Cases in this edition contained slightly fewer vertical initiatives, and more emphasis was placed on innovative financing techniques like targeted cash transfers. Ultimately, the new edition offered a similar definition of success: the highest impact on one or more health outcomes, ideally supported with cost-effectiveness studies.^87^ The successes taught in the recent *Millions Saved* volume are now required readings at more than 60 universities worldwide,^88^ and are seen as teachable, stand-alone lessons for rolling out global health programmes.

Other major global health funders began to embrace the impact framework well before the Center for Global Development and the Gates Foundations’ highly publicised work.^89^ For instance, in the late 1980s, the Bank and the Clark Foundation partnered on an empirical study to identify health successes in the developing world. They defined success as sustained achievement of programme objectives, as documented by clear evidence and international recognition. The study was only able to identify seven major global health successes in Sub-Saharan Africa, one of which was the OCP. It concluded that successful programmes are technology-driven, efficiently managed and community-oriented, run ‘almost like a well-trained guerrilla unit’. Successes were stories that could stand alone, were demonstrable through distinct evidence, and could form a lessons base for future interventions, irrespective of the precise location, time (historical period) or actors involved in these future endeavours.^90^ The World Bank Independent Evaluation Group’s recent studies, based on its ‘What Works’ framework, have also looked for factors that influence the success or failure of Bank projects, on the basis that an entire programme can be a discrete success (or failure).^91^

*The marketing framework:* Like the impact/evidence-based framework, the marketing framework emphasises the importance of empirical evidence for defining success. However, it addresses some of the criticisms of using metrics in isolation and views success as inseparable from a health intervention’s socio-political climate. The marketing perspective instead places a strong emphasis on political precedent; a programme’s status as a ‘success story’ is due at least as much to its being showcased and marketed as to its actual measured impact. In such a way, success is relative and can change (even after a health programme’s end). A programme may fail in some (or many) of its stated objectives, but it may still set a political precedent and transform into a long-term success.

The health policy scholar Jeremy Shiffman has worked within this framework. Shiffman argues that ‘policy images’ (conceptualisations of the health problem or solution) and ‘policy venues’ (shifts in decision-making actors and institutions) are critical for building global health priority.^92^ The success of the programme was not its impact per se, but the *perception* that it was successful and the *precedent* that it set for future similar disease control efforts. The marketing model therefore places a strong analytical emphasis on the tools that global health actors use to construct policy images because these images have the power to change perceptions of success. In her analysis of the Bank’s role in nutrition, Devi Sridhar points to how the Bank’s marketing of the Tamil Nadu Integrated Nutrition Project allowed it to serve as a ‘model for success’, despite internal confusion about how to operationalise cost-effective analyses for social sector projects.^93^ Following this logic, Sophie Harman has argued that the World Bank’s Multi-Country AIDS project was an ‘ideological’ success by serving as a model for future programmes like UNAIDS and the Global Fund to Fight HIV/AIDS, Malaria and Tuberculosis, while also being ‘accompanied by distinct failure’ to meet its objectives.^94^

The marketing framework has been particularly important to sponsoring agencies seeking to showcase their development assistance successes in Africa since the 1980s, amidst considerable publicity of regional ‘failures’. A WHO Commission on Macroeconomics and Health (2001) literature review, for instance, concluded that Africa was depicted the most negatively of all regions,^95^ while a study of Africa’s depiction in the *New York Times* found that 73 per cent of all articles from 1955 to 1995 had a negative image of African politics and society (the Bank featured in a significant proportion of these articles).^96^ Seen in this light, showcasing the OCP provided a vehicle for balancing the image of the Bank, particularly when it faced structural adjustment-related criticisms in the late 1980s and 1990s. Such narrative marketing techniques have been used by other institutions of power. Davide Rodogno and Thomas David have described, for instance, how the WHO developed tropes for humanitarian narratives (or ‘alleged success stories’) in the 1950–70s, which were ‘dramatically narrated and visually staged’.^97^

*The constructivist framework* *:* Building on the idea of success as appearance, this framework analyses success as the product of its historical era and influential global health actors. Like the marketing framework, it emphasises that success can *only* be understood by analysing a programme’s precise location, timing and actors. It defines success as *entirely constructible* by powerful actors like the Bank and Gates Foundation, to showcase programmes that fit with their entrenched institutional ethos.

For example, Sanjoy Bhattacharya argues that the WHO’s histories of smallpox eradication often depict the transfer of expertise as unidirectional, universal and uncontested, based on a selective use of metrics and primary sources. He contends that complex issues like societal resistance are ‘frequently downplayed in tomes seeking to celebrate the contributions of individuals, institutions and national governments’, which help organisations like the WHO institutionalise their power.^98^ Katerini Storeng similarly points to the constructible nature of metrics, by criticising policymakers’ tendencies to overlook programmes’ complexity when they focus on measuring programme impact, in the context of the GAVI Alliance and health system strengthening (HSS).^99^ By promoting HSS successes using its internally-produced metrics, she argues that GAVI is able to silence critics of its narrow focus on vaccines. Birn’s writings on success in global health form the heart of the constructivist framework. In her 2009 article on the historical stages of international health success, she states that the global health community has been in a stage of ‘uncontested success’ since 1985, reflecting the business models of dominant actors.^100^ Birn’s 2011 article on ‘Small(pox) success?’ expands upon these stages, using a case study of global smallpox control to explore how success becomes un-contestable. In doing so, she underscores that even the success of smallpox eradication, widely considered to be ‘the single greatest public health success in history… a quintessential global health parable’, can and should be questioned.^101^

The marketing and constructivist frameworks share many features. Both frameworks do not view success as an endpoint; a programme can never reach a point at which it is a stand-alone ‘success’, because when venues change, definitions (and designations) of success also change. The distinguishing characteristic of the constructivist framework is its heavy emphasis on institutional power. Within the marketing framework, metrics are at least somewhat evidence-based, and it is the *dissemination of these metrics* that determines a programme’s success status. In contrast, constructivists argue that ‘evidence-based’ *data and metrics themselves bend* epistemologically to the ethos of institutions and their policy images. Data can never stand on its own or be self-evident.

The socio-economic metrics that the Bank used to portray the OCP as a success story were the product of significant, typically *under-acknowledged assumptions* and were *selectively operationalised*. I argue that they—and the narratives based upon them—are *constructible*. Table 2 summarises OCP socio-econometric metrics and how they varied dramatically over the duration of the OCP. While the numbers underpinning many assumptions, like the value of the marginal output of labour, number of individuals at risk for disease and land differential, did not come out of a magic hat, they were also often crude estimates based on limited data from single countries.

**Table 2.** Estimates for the rate of return, net present value and land-freed values show significant variation

Metric	Value	Study
Rate of return	10 per cent	Preparatory Mission, 1973
	−6 to 69 per cent	Younger and Zongo, 1989
	11–13 per cent	Benton and Skinner, 1989
	63 per cent (labour only)	Benton and Skinner, 1989
	18–21 per cent	Kim and Benton, 1995
Net present value	$20.5 million to $1,137.6 million (labour)	Economic Review Mission, 1978
	$30 million to $1,137 million (labour), $−123 million to 315 million (land)	Younger and Zongo, 1989
	$485 million to $3,729 million (labour and land)	Kim and Benton, 1995
Land freed for cultivation	65 million hectares (7 countries)	Preparatory Mission, 1973
	5.06, 13.4, or 15.4 million hectares (7 countries)	Economic Review Mission, 1978
	15 million hectares	USAID Impact Evaluation, 1986
	9.9 or 18.2 million hectares (11 countries)	Younger and Zongo, 1989
	25 million hectares (11 countries)	Kim and Benton, 1995

When combined with the fact that there was no gold standard for the discount rate and time horizon to use for a rural health programme, the Bank had significant leeway in how it chose to calculate metrics. Kim and Benton’s heavily cited rate of return and land available for resettlement, for instance, raise as many questions as answers about how to model socio-economic success for disease control programmes. Was it fair to consider 25 million hectares of land to be available, based largely on a decade-old, contested Bank study? How realistic was it to extrapolate from limited data often collected in the early-mid 1980s that 85 per cent of this rural land—which often lacked roads and access to basic social services—would be settled shortly after the end of the OCP? What about the costs associated with settling the land, which would be borne outside the OCP? Should these ‘extra-programmatic’ costs be factored into the OCP’s rate of return because the rate of return primarily reflected the potential land value? And, if extra-programmatic costs were included in the cost-benefit analyses, should they also include future epidemiological maintenance activities? Given that the land benefits changed so dramatically depending on the time horizon used, did cited rates of return even mean anything, if the authors citing them did not specifically stipulate the time frame? Perhaps most importantly, if including extra-budgetary costs or reducing the land estimates made the rate of return drop below the standard lending threshold of 5–10 per cent, would it mean that health programmes like the OCP should not be prioritised for investment?

In their analysis of the ‘limited’ and ‘broadly positivist’ literature on the development of health system metrics during the twentieth century, Martin Gorsky and Christopher Sirrs identify three major types of stakeholders involved in creating and disseminating international and global health statistics. These include the individuals and institutions who commission or publicise indicators (‘promulgators’); create and maintain the supply of indicators (‘providers’, often subject-matter experts); and serve as subjects of measurement or funders who react to the metrics (‘users’).^102^ Jean-Paul Gaudillierè and Camille Gasnier have similarly explained how DALY-related metrics (particularly the Global Burden of Disease) can reveal a ‘new political order’ and offer a lens to interrogate the political economy of global health.^103^

In the case of the OCP, the Bank’s role as both a provider and promulgator of economically grounded health metrics reflects what Tichenor et al. have described as its epistemic, discursive and institutional power.^104^ The OCP was included in the 1993 *World Development Report* to showcase how combining DALYs and cost-effectiveness could help prioritise diseases associated with poverty^105^ and in recent years econometrics provided by the Bank have been used for ‘investment cases’ for NTDs by development agencies;^106^ Harvard University has called NTDs ‘Big Bang for the Development Buck’;^107^ and the Bank has deemed NTDs ‘one of the best buys in public health’.^108^ Since at least the mid-1990s, the Bank has depicted the programme’s success as finite and incontestable, based on clear, simple metrics (like the number of hectares of land saved and number of people that could be fed from this land).^109^

Randall Packard has described a strikingly similar pattern for malaria control metrics construction.^110^ Packard contends that, from the 1950s to the 1980s, public health professionals and multilateral institutions struggled to find appropriate socio-economic data for precise quantitative studies of malaria’s impact. With the launch of the *World Development Report* in 1993, he states that ‘the Bank ignored the methodological concerns that economists and demographers had raised since the 1960s’ about malaria economic modelling. In the 1990s and 2000s, high rates of return from ‘armchair regression analyses’ were produced by economists like Jeffrey Sachs.^111^ According to Packard, instead of providing new methods for measuring socio-economic impact, these studies glossed over many of the prior long-standing challenges with calculating health-related rates of return. Like the citations of Kim and Benton’s 1995 study, ‘the vast majority of references to Sachs’ research are not by economists. Instead they appear in medical publications in which his articles are used uncritically to legitimise further research’ on various aspects of malaria. As Packard puts it, ‘for this large scientific and public health audience, the value of Sachs’ work clearly lies in bolstering the arguments of those who cite him’.^112^ In the case of malaria, this audience included the new Roll Back Malaria initiative in the late 1990s and the Global Fund in the early 2000s.

## Conclusion: (Constructively) Countering the (Impact-Based) Success Cartel

 In addition to metrics, the narratives that they are weaved into can be actively constructed. In the case of the OCP, the Bank’s Information and Public Affairs department actively looked for success stories in the 1980s, and *Reader’s Digest* contacted the Bank with a similar request for the health sector.^113^ Although the Bank’s onchocerciasis coordinator admitted that he was ‘doubtful whether OCP provides a framework for generalizing about Bank effectiveness or current development concerns’, he suggested that the OCP success story could well demonstrate the ‘payoffs of long-term donor commitment and well-coordinated aid’. He also pitched the OCP as a ‘useful counterweight to the Bank’s sometimes “hard guy” image’.^114^ Bank leaders also recognised the value of charisma for initiating and maintaining support from the donor community for onchocerciasis.^115^ In a videotaped press interview with an ageing Robert McNamara from the 1990s, for instance, he cites the 25 million hectares figure and is seated next to a replica of the onchocerciasis statue.^116^ Yet, according to a file within the WHO Archives, the most famous photograph (Figure 1) may have been staged by a photographer in the late 1960s a few years before the OCP was launched, in a region outside of the future OCP control zone.^117^

Arguing that the OCP’s (and Roll Back Malaria’s) success was constructed does not imply that the programmes were not successful in other specific ways. Similarly, challenging the objectivity of some programme metrics does not mean that all metrics are based on ‘fake data’ or should be discarded. For instance, the OCP’s population health metrics relied on solid data collection in indicator villages throughout the OCP area, which showed a significant decline in disease transmission by the early 1980s. They indicate that the programme *did* have a strongly positive impact on the burden of regional onchocerciasis.

Instead, the OCP example highlights how success narratives are rarely passively formed, often overlook the nuanced legacies of global health programmes, and always represent entrenched ethos and power of global health actors. This trend extends well beyond the World Bank. For instance, Harman has argued that the ‘Bill Chill’ (Gates Foundation influence) stifles in-depth programme analyses and criticisms, and Yogesh Rajkotia has described how competition for limited development assistance for health incentivises a ‘success cartel’ in global health, characterised by the embellishment of positive aspects of programmes and neglect of negative aspects.^118^

If success narratives can be built on pliable metrics by powerful actors like the Bank and Gates Foundation (through, as Birn has put it, their ‘investing in health’ ethos),^119^ what is the pathway forward for strategic global health planning and research? A first step is avoiding declarations of whole *programmatic* success and instead asking *specific questions* about what worked, what did not and under which political or cultural contexts. In the case of the OCP, querying how indicator villages can best be set up for epidemiological monitoring in rural areas, or what the programme reveals about how to standardise variables (like discount rates) across socio-economic metrics for disease control, would provide a better pathway for replicating the programme’s success*es* (and avoiding its failure*s)* than looking at the programme as a collective. Disrupting success narratives and turning a critical historical eye to the power structures and systems of ideas that underpin metrics can help to counter the success cartel. It can also challenge the epistemic colonialism^120^ and the marginalisation of scholarship that is focused on interrogating power^121^ that is all too pervasive within global health history.

## Footnotes

1 s.a., ‘Forty years later: the extraordinary river blindness partnership sets its sights on new goals’, *World Bank Group News* (3 July 2014).

2 Frances Vieta, ‘River blindness’, *UN Chronicle* 1 January 1998.

3 Boakye Boatin, ’The Onchocerciasis Control Programme in West Africa (OCP)’, *Annals of Tropical Medicine & Parasitology* 102 sup1 (2008), 13–17.

4 Peter Hotez, ‘Enlarging the “audacious goal”: elimination of the world’s high prevalence neglected tropical diseases’, *Vaccine* 29 (2011), D104–110.

5 By 1987, the OCP Documentation Centre had processed more than 1,700 articles or reports and 1,324 book references. See Onchocerciasis Control Programme, *Joint Programme Committee, eighth session, Rome, 30 November—3 December 1987* (Geneva: WHO, October 1987).

6 APOC, *Curriculum and training module on the Community-Directed Intervention strategy for faculties of medicine and health sciences*, 2^nd^ ed (Geneva: WHO, 2012), 26.

7 Examples of newspaper articles include James Brooke, ‘Ancient scourge loses ground in West Africa’, *Special to the New York Times,* 3 September 1987; Erik Eckholm, ‘River blindness: conquering an ancient scourge’, *New York Times,* 8 January 1989; Beth Duff-Brown, ‘Taming the lion’s look: world health agency trying to stop river blindness’, *Associated Press,* 22 October 1995.

8 Ministere des affairs étragères, *Half a century of onchocerciasis control in West Africa: France’s commitment* (Paris: Institut de recherché pour le developpement, 2003).

9 Scopus, Web of Science, PubMed, ProQuest and Google Scholar were searched in June 2017, using the keywords ‘Onchocerciasis Control Program*’ and ‘success’. To be included, articles/books/papers had to have the success directly referring to the OCP.

10 Aehyung Kim and Bruce Benton, *Cost-benefit analysis of the Onchocerciasis Control Program (OCP)* (Washington DC: World Bank, 1995).

11 Steven D. Younger and Jean-Baptiste Zongo, ‘West Africa: The Onchocerciasis Control Program’, in *Successful development in Africa: case studies of projects, programs, and policies* (Washington DC: World Bank, 1989), 44.

12 World Bank Group Archives (WBGA), ‘Oncho AV for Janelle Winters’, digitised September 2018.

13 David Bannister, ‘Willful Blindness: sleeping sickness and onchocerciasis in colonial Northern Ghana, 1909–1959’, *Social History of Medicine* 35:2 (2022), 635–660. See also John Hunter, ‘Progress and concerns in the World Health Organization Onchocerciasis Control Program in West Africa’, *Social Science and Medicine* 15:2 (1981), 261–275.

14 See WHO, *Fourth report on the world health situation, 1965–1968* (Geneva: WHO, 1971), 88–90.

15 Randall Packard, *A history of global health: Interventions into the lives of other peoples* (Baltimore: Johns Hopkins University Press, 2017).

16 Daniel Speich Chassé. ‘When economics went overseas: epistemic problems in the macroeconomic analysis of late colonial Africa’, in Martin Lengwiler et al. (Eds), *Science, Africa and Europe* (London: Roudledge, 2018), p. 237–255.

17 Jesse Boardman Bump, *The Lion’s Gaze: African river blindness from tropical curiosity to international development,* doctoral thesis (Baltimore: 2004, Johns Hopkins University Press).

18 For more on the McNamara administration’s transition to a human capital perspective, see Patrick Allen Sharma, *Robert McNamara’s other war: the World Bank and international development* (Philadelphia: University of Pennsylvania, 2017), 54–75; Devesh Kapur et al., *The World Bank: its first half century, Vol. 1* (Washington DC: Brookings Institution Press, 1997), 51–55, 215–330; Martha Finnemore, ‘Redefining Development at the World Bank’, in Frederick Cooper and Randall Packard (Eds.), *International development and the social sciences: essays on the history and politics of knowledge* (Berkeley: University of California Press, 1997), 203–227.

19 Some senior staff at the Bank, USAID, UNDP, FAO and WHO were interested in quantifying the link between health and economic development. Representatives from these agencies began to meet about onchocerciasis and economic models in 1966, at the suggestion of the WHO and USAID. See Bump, *Lion’s Gaze*, 332, 335, 346–347.

20 WHO Archives (WHOA), O8-372-3, Leonard Rist to N. Ansari, 8 February 1967.

21 United Nations Archives (UNA), s-0553-14-6, Leon Segal, Memorandum, 3 April 1972.

22 See WBGA, 1082632, ‘From the President, control of oncho in Western Africa’, 16 April 1973; WBGA, 1434979, ‘IBRD press release—grand strategy against river blindness: Volta river basin target for $120 million campaign’, 5 September 1973.

23 See, for instance, Mollie Fair, *From population lending to HNP results: the evolution of the World Bank’s strategies in Health, Nutrition and Population* (Washington DC: World Bank, 2008); World Bank, *Health sector policy paper* (Washington DC: World Bank, 1980).

24 World Bank, *Onchocerciasis Fund Agreement* (Washington DC: World Bank, 1975).

25 WBGA, 10300662, ‘Draft, oncho control in the Volta River basin area, preparation of master plans for economic development of cleared land and of investment project proposals’, 9 August 1974.

26 WBGA, 30339647, Samuel C. Adams, ‘EDAP 2^nd^ meeting, Paris, October 1–2’, 1976.

27 WBGA, 10300662, ‘Draft, oncho control’.

28 See WHOA, O8-372-3, Peter F. Betsche to John Lawton, [1972].

29 The gross investment required for full development of the Sahel over thirty years was estimated at $15 billion. WBGA, 30339647, Samuel C. Adams, ‘EDAP 2^nd^ meeting’.

30 For more on human capital at the Bank in the 1970s–1980s, see David E Bloom and David Canning, ‘Population health and economic growth’, in *Health and growth* (Washington DC: World Bank, 2009), 53–75; Devi Sridhar, *The battle against hunger: choice, circumstance and the World Bank* (Oxford: Oxford University Press, 2008), 89-94.

31 WBGA, 1082632, E. Peter Wright to R. McNamara, ‘Paris Meeting on Riverblindness’, 2 July 1974.

32 See WBGA, D81087, Huntley Consultants, ‘SED studies in the OCP area—final report’, 1986.

33 WBGA, 1170231, FAO Agricultural Services Division, ‘An introduction to socio-economic development planning and technical assistance to the onchocerciasis freed zones of the Volta River basin area (OCP)’, May 1976.

34 See WBGA, 1170231, ‘Reporting on preliminary mission to countries concerned with the onchocerciasis projects’, June 1975.

35 WBGA, 1170231, Robert Smith, ‘Report on visit to Onchocerciasis Control Programme, West Africa, March 30–April 11’, April 1976.

36 WBGA, 30339648, Stephen Denning to André Gué, ‘EDAP options’, 21 November 1977.

37 Food and Agriculture Organization Archives (FAOA), 10-AGD-405, T.S.E. Aribisala to Oncho Task Force Members, ‘Proposal to review/revise the PAG mission report in the light of progress and developments in the Onchocerciasis Control Programme’, 2 December 1977.

38 Elliot Berg et al., *Accelerated development in Sub-Saharan Africa: an agenda for action* (Washington DC: World Bank, 1982).

39 WBGA, 1082632, Roger Chaufournier to Robert McNamara through Ernest Stern, 21 September 1978.

40 Younger and Zongo, ‘West Africa’, 27–56.

41 This reduction was based on soil impurities, and lack of water, firewood and necessary infrastructure. In proposed extension zones, Berg estimated that 48,440 additional km^2^ would be cultivatable. *Ibid,* 34.

42 WBGA, 1434979, Bilsel Alisbah to Roger Chaufournier, ‘Onchocerciasis Control Program JCC meeting of December 5–8’, 12 December 1978.

43 FAOA, 10-AGS-239, February-June 1979, T.S.B. Aribisala to D.F.R. Bommer, ‘Back-to-office report after attendance in Paris at the 5th meeting of EDAP (11-13 September 1978)…’, 9 February 1979.

44
*Ibid.*

45 Stein H, *Beyond the World Bank agenda: an institutional approach to development* (Chicago: University of Chicago Press, 2008), 207–248.

46 WBGA, 30303429, J.P. Dailly to Files, ‘Onchocerciasis Control Programme: visit to donor countries back-to-office report’, 16 November 1983.

47 WBGA, 1300674, J.P. Dailly to Files, ‘Back-to-office report, donors meeting 1984’, 2 November 1984.

48 WBGA, 0146782, Nick Prescott to J. Westford, ‘Interim report of the Gordon Smith independent commission on the Onchocerciasis Control Program’, 7 January 1983.

49 Anne Mills, ‘Reflections on the development of health economics in low-and middle-income countries’, *Proceedings of the Royal Society B: Biological Sciences* 2014, 281:1789, 20140451.

50 Less ideally, a cost-effectiveness analysis could also help programme leaders choose the variant with the minimum cost per some unit of effectiveness, such as a quality-adjusted life year (QALY).

51 Andre Prost and Nicholas Prescott, ‘Cost-effectiveness of blindness prevention by the Onchocerciasis Control Programme in Upper Volta’, *Bulletin of the WHO,* 1984, 62:5, 795.

52 See Martin S Feldstein et al., ‘Resource allocation model for public health planning’, *Bulletin of the WHO,* 1973, 48, 5; George W. Torrance, ‘Health status index models: a unified mathematical view’, *Management Science* 1976, 22, 990–1001.

53 Prost and Prescott, ‘Cost-effectiveness’. See also Younger and Zongo, ‘West Africa’.

54 Prost and Prescott, ‘Cost-effectiveness’.

55 WBGA, 1300674, ‘Briefing: OCP donors’ seminar’, [1985].

56 WBGA, D81087, Huntley Consultants, ‘SED studies’.

57 Suitability was defined by the Preparatory Mission report based on water availability (i.e., of the 276,000 km^2^ of land made available by the OCP, about 150,000 km^2^ was located in the 800–1,000 mm/year rainfall zone). See OCP, *WHO independent commission of the long-term prospects of the Onchocerciasis Control Program, final report* (Geneva: WHO, 1981), c-4.

58 This further assumes that an average family of ten people eats two tons of cereal grain a year to meet basic caloric needs, and nothing else, and that about 800 kilograms of sorghum could be realistically grown per hectare on the OCP lands (totalling about 2 million tons of sorghum per year).

59 WBGA, 809925, ‘CSA report on mechanisms for promoting SED in oncho controlled zones’, 24 October 1986.

60 Based on Younger and Zongo, ‘West Africa’.

61 These included a high value of labour, coupled with any rate of labour productivity growth (one or three per cent), a low discount rate (five per cent) and a 50-year time horizon. A 25-year time horizon never produced an acceptable rate of return under the most optimistic assumptions of income and productivity growth, nor did a high (15 per cent) discount rate.

62 J.P. Herouvet et al., *Peuplement des vallees proteges de l’onchocercose après dix ans de lutte anti-vectorielle au Burkina Faso* (Ouagadougou: OCP, 1984).

63 Younger and Zongo, ‘West Africa’, 39.

64 WBGA, 1300688, Bruce Benton and Elizabeth Skinner, ‘Cost-benefits of onchocerciasis control (draft)’, September 1989.

65
*Ibid.* They argued that, in the case of health in a developing country, it is appropriate to choose a lower ‘social’ discount rate (5 per cent), because benefits to the rural poor are more highly valued than returns benefitting other segments in the population and health programmes are often designed for long-term progress.

66 Benton and Skinner used the mission’s high estimate and added Bank internal estimates of the land available in the western extension and southern extension, to reach a grand total of 25 million hectares. They give the sources for this data as the ‘Berg report’ (referring to the Economic Review mission, 1978) for the original and southern extension, and ‘other World Bank estimates’ for the Western extension.

67 When calculating the internal rate of return for labour, Benton and Skinner arbitrarily chose to consider only 15 million hectares of land suitable for cultivation, to be ‘conservative’.

68
*Ibid.*

69 OCP, *External review of the Onchocerciasis Control Programme* (Geneva: WHO, 1990), 21.

70
*Ibid,* 16.

71 Vincanne Adams, *Metrics: what counts in global health* (Chapel Hill: Duke University Press, 2016),

72 Katherine E Kenny, ‘The biopolitics of global health: life and death in neoliberal time’, *Journal of Sociology*, 2015, 51(1), 9–27.

73 Stein, *Beyond the World Bank*, 219-220, 22.

74 WBGA, 1428489, Cost-benefit analysis presentation slides, [November 1994].

75 Kim and Benton, *Cost-benefit analysis.*

76
*Ibid.* Following an s-shaped curve function; once the incidence of onchocerciasis approached zero (about 8 years after vector control operations began in an area), use of this new land would begin slowly and then proceed faster, up to a certain level (the 25 million hectares) when it was fully cultivated. In their net present value and rate of return calculations, the authors also largely arbitrarily decided that it would be more realistic if 85 per cent (rather than 100 per cent) of the land was eventually cultivated.

77 Tim Evans, ‘Socioeconomic consequences of blinding onchocerciasis in West Africa’, *Bulletin of the WHO,* 1995, 73:4, 495–506.

78 Anne-Emanuelle Birn, ‘The stages of international (global) health: histories of success or successes of history?’, *Global Public Health,* 2009, 4:1, 50–68; Anne-Emanuelle Birn, ‘Small(pox) success?’, *SciELO Public Health,* 2011, 591–596.

79 Moon outlines eight types of power (physical, economic, structural, institutional, moral, discursive, expert and network). Suerie Moon, ‘Power in global governance: an expanded typology from global health’, *Globalization and Health*, 2019, 15(1), 1–9.

80 Marlee Tichenor et al., ‘Interrogating the World Bank’s role in global health knowledge production, governance and finance’, *Globalization and Health*, 2021, 17, 1–12.

81 See Bill & Melinda Gates Foundation, ‘Press room: Campaign to highlight success of U.S. global health investments’, 2009 (online), <https://www.gatesfoundation.org/media-center/press-releases/2009/09/campaign-to-highlight-success-of-US-global-health-investments> [accessed 18 June 2019].

82 Ruth Levine and the What Works Working Group, *Millions saved: proven successes in global health* (Washington DC: Center for Global Development, 2004); Center for Global Development, *CGD brief—millions saved: proven successes in global health* (Washington DC: CGD, March 2007).

83 Six (of 16) members of the What Works Working Group, which selected the initial case studies for *Millions saved*, were former long-term Bank staff, and another two were former Bank advisors or consultants. Center for Global Development, ‘About: the What Works Working Group’ (online), <https://www.cgdev.org/page/about-0> [accessed 18 June 2019].

84 Levine and What Works, *Millions saved*; Levine, *Case studies*; Amanda Glassman and Miriam Temin, *Millions saved: new cases of proven success in global health* (Washington DC: Center for Global Development, 2016). See also Center for Global Development, ‘Millions saved’ (online), <http://www.millionssaved.cgd.org> [accessed 18 June 2019].

85 Projects had to be long-term and lasting at least 5 years to be included. Levine, *Case studies*, Chapter 1.

86 Levine and What Works, *Millions saved*; Levine, *Case studies*.

87 Glassman and Temin, *Millions saved*.

88 Center for Global Development, ‘Impacts and influence: millions saved’ (online), <https://www.cgdev.org/impacts-and-influence/millions-saved> [accessed 18 June 2019]; Center for Global Development, *Instructor guide—Millions saved: proven successes in global health* (Washington DC: CGD, s.d.).

89 The Bank’s Onchocerciasis Coordinator, Bruce Benton, was asked to go through the first volume of *Millions saved* (WBGA Oral History Program, *Transcript of an interview with Bruce Benton* [Washington DC: World Bank, 2006], 86), and the 2004 *Millions saved* edition was disseminated to leaders throughout the development and health field, including at the United Nations (UNA, s-1096-0103-06-00007, Nancy Birdsall to Colleague, 30 November 2004).

90 Bernhard Liese et al., *Organizing and managing tropical disease control programs: lessons of success* (Washington DC: World Bank, 1991).

91 See Independent Evaluation Group, *Results and performance*, 171.

92 Jeremy Shiffman J and Stephanie Smith, ‘Generation of political priority for global health initiatives: a framework and case study of maternal mortality’, *Lancet,* 2006, 370:9595, 1370-79; Jeremy Shiffman et al., ‘The emergence of global disease control priorities’, *Health Policy and Planning,* 2002, 17:3, 225–34.

93 Sridhar, *The battle against hunger*, 5–6, 85–98.

94 Sophie Harman, ‘The World Bank: failing the multi-country AIDS program, failing HIV/AIDS’, *Global Governance,* 2007, 13, 485–492.

95 Anna Breman and Carolyn Shelton, ‘Structural adjustment and health: a literature review of the debate, its role-players and presented empirical evidence’, *CMH Working Paper Series, No. WG6:6* (Geneva: Commission on Macroeconomics and Health, 2001).

96 Peter J Schraeder and Brian Endless, ‘The media and Africa: the portrayal of Africa in the *New York Times* (1955–1995)’, *Issue: A Journal of Opinion,* 1998, 26:2, 29–35.

97 Davide Rodogno and Thomas David, ‘All the world loves a picture: the World Health Organization’s visual politics, 1948–1973’, in Heide Fehrenbach (Ed.), *Humanitarian photography: a history* (New York: Cambridge University Press, 2015), 223–248.

98 Sanjoy Bhattacharya, ‘Reflections on the eradication of smallpox’, *Lancet,* 2010, 375:9726, 1602–03.

99 Katerini Storeng, ‘The GAVI Alliance and the “Gates approach” to health systems strengthening’, *Global Public Health,* 2014, 9:8, 865–79.

100 Birn, ‘The stages’.

101 Birn, ‘Small(pox) success?’.

102 Martin Gorsky and Christopher Sirrs, ‘World health by place: the politics of international health system metrics, 1924-c. 2010’, *Journal of Global History* 2017, 12, 361–385.

103 Jean-Paul Gaudillier*è* and Camille Gasnier, ‘From Washington DC to Washington state: the global burden of diseases data basis and the political economy of global health, in Sabina Leonelli and Niccolo Tempini (Eds.), *Data journeys in the sciences* (New York: Springer, 2020), 351–369.

104 Tichenor et al., ‘Interrogating’.

105 World Bank, *World development report 1993: investing in health* (New York: Oxford University Press, 1993), 158–159, 167–168.

106 Harvard University, ‘Neglected tropical diseases: progress and challenges for the post-2015 development era’, *Science in the News* (31 December 2014).

107 For instance, the OCP’s 25 million hectares and cost per person protected was used for the Bank’s NTD investment case. See Harvard University, ‘Neglected tropical diseases’ (2014); Donald Bundy, ‘Neglected diseases of neglected people: the case for investing in neglected tropical diseases’, *World Bank Blogs* (online), 14 February 2013 [accessed 15 February 2017].

108 Bundy, ‘Neglected diseases’.

109
*Ibid.*

110 Randall Packard, ‘“Roll back malaria, roll in development”? Reassessing the economic burden of malaria’, *Population and Development Review,* 2009, 35:1, 53–87.

111 See, for instance, John Luke Gallup and Jeffrey D. Sachs, ‘The economic burden of malaria’, *American Journal of Tropical Medicine and Hygiene,* 2001, 64:1, 85–96.

112 Packard, ‘Roll back’.

113 WBGA, 30304887, Henri Bretaudeau to Michael Carter, ‘Reader’s Digest’, 16 September 1986.

114 WBGA, 30348069, Bruce Benton to Caio Koch-Weser through Jeannie Murphy, ‘Your question re. publicity for success stories’, 25 July 1986.

115 World Bank, *Transcript of an interview with Bruce Benton*, 42.

116 WBGA, ‘Oncho AV for Janelle Winters’, digitised September 2018.

117 See WHOA, O8-370-4, C.D. Ginger to David A. Denham, ‘Request for photograph’, 11 January 1993.

118 Yogesh Rajkotia, ‘Beware of the success cartel: a plea for rational progress in global health’, *BMJ global health*, 2018, 3:6, e001197.

119 Birn, ‘The stages’.

120 Eugene Richardson, *Epidemic illusions: on the coloniality of global public health* (Boston: MIT Press, 2020), 7, 10, 46.

121 Hani Kim et al., ‘A critical assessment of the ideological underpinnings of current practice in global health and their historical origins’, *Global Health Action*, 2019, 12:1, 1651017.

## Notes

Janelle Winters is a historian of global health at the University of Manchester and the University of Oxford. Her current research explores neglected tropical diseases markets, access to drugs and evidence production in global health and humanitarian medicine since the 1980s. She is an affiliate researcher with the University of Edinburgh’s Global Health Governance Programme, where her doctoral research explored the historical influence of the World Bank on global health. Recently, she served as the director of a global pandemic preparedness study at the U.S. National Academy of Sciences.

